# Cross-cultural translation, reliability and validity of the Thai version of the Patient‑Reported Outcomes Measurement Information System (PROMIS) Parent Proxy Upper Extremity Short Form 8a in children with congenital upper extremity anomalies

**DOI:** 10.1186/s12955-023-02141-x

**Published:** 2023-06-19

**Authors:** Pobe Luangjarmekorn, Pongsathorn Sitthisen, Vanasiri Kuptniratsaikul, Pravit Kitidumrongsook

**Affiliations:** Department of Orthopaedics, Faculty of Medicine, Chulalongkorn University, King Chulalongkorn Memorial Hospital, Patumwan, Bangkok, 10330 Thailand

**Keywords:** PROMIS, Parent proxy, Upper extremities, Thai, Translation

## Abstract

**Background:**

The PROMIS Parent Proxy Upper Extremity Short Form 8a version 2 (PROMIS Parent Proxy UE-SF) is one of the most commonly used self-assessment questionnaires for evaluating function in children with congenital upper extremity anomalies. However, this English questionnaire is difficult for Thai parents to complete. The purpose of this study is to translate the PROMIS Parent Proxy UE-SF into Thai and test its reliability and validity.

**Methods:**

The PROMIS Parent Proxy UE-SF was translated into Thai using FACIT translation methodology. This version and the Thai version of the Michigan Hand Questionnaire (Thai-MHQ) were used to evaluate 30 Thai children with different types of congenital upper extremity anomalies. The reliability and validity of the Thai-PROMIS Parent Proxy UE-SF were evaluated by test-and-retest with the intraclass correlation coefficient (ICC) and Cronbach’s alpha coefficient. Correlations between the Thai-PROMIS Parent Proxy UE-SF and Thai-MHQ were analysed by Pearson’s correlation coefficients.

**Results:**

The children’s mean age was 4.47 ± 2.08 years (range 1–9 years). The main diagnoses included thumb duplication (11 children), syndactyly (4 children)^4^, congenital trigger thumb (3 children) and obstetric brachial plexus palsy (3 children). The children’s parents completed the questionnaires, taking 164.23 ± 22.58 s for the Thai-PROMIS and 337.8 ± 49.37 s for the Thai-MHQ. The test-retest reliability of Thai-PROMIS evaluated by ICCs, was 0.9909 (good reliability), and the Cronbach’s alpha of all items was 0.923. The Pearson’s correlation coefficient between the Thai-PROMIS and Thai-MHQ showed a strong correlation with Domain 2 (activities of daily living, r = 0.7432) and a moderate correlation with the overall Thai-MHQ score (r = 0.699).

**Conclusions:**

The Thai-PROMIS Parent Proxy UE-SF is a valid, reliable and easy-to-use patient-reported outcome measure for assessing function in children with congenital upper extremity anomalies.

## Background

Congenital upper extremity anomalies usually affect several functions and lead to a delay in children’s development, daily life activities and education [1]. A variety of anomalies need specific treatments, including surgical and nonsurgical interventions. Treatment should start at birth and continue until the children are grown. A tool to assess upper extremity functions in children would be helpful in guiding patients, parents and caregivers in planning, monitoring and evaluating results before and after treatment. Self-report questionnaires are a good and easy way to evaluate and monitor progress during treatment. Tools that are easy to use and suited to young patients should be available for use.

Several standardized measures have been developed in recent years with the intent to quantify the burden of disease and the response to medical treatments from the patient’s perspective. These commonly used patient-reported outcome measures [2] include the Disabilities of the Arm, Shoulder and Hand (DASH) questionnaire [3], the Brigham and Women’s Hospital Carpal Tunnel Questionnaire (CTQ) [4], the Patient-Rated Wrist Evaluation (PRWE) questionnaire [5], and the Short-Form-36 (SF-36) [6] and the Michigan Hand Outcome Questionnaire (MHQ) [7]. Currently, there are some Thai-translated self-report questionnaires available for use, such as the Thai-MHQ, Thai-DASH and Thai-PRWE [8–11]. However, none are designed specifically for young children or parent proxies of these children.

There are many tools available in the English language that are used to evaluate the upper extremities of children, such as the Pediatric Outcomes Data Collection Instrument (PODCI), ABILHAND-KIDS and Patient‑Reported Outcomes Measurement Information System (PROMIS). The PODCI assesses all domains, including function, ability to perform the activities of daily living (ADLs), appearance, emotion, pain and social interaction [12]. However, the instrument takes a lot of time to complete, which may not be appropriate for young children. The ABILHAND-KIDS is a measure of manual ability for children with upper limb impairments. The scale measures a person’s ability to manage daily activities that require the use of the upper limbs. This questionnaire evaluates 21 items according to three response levels: impossible, difficult and easy [13]. The PROMIS has a variety of domains and is available in both full and short forms for evaluating the specific domains that are of interest to reduce the time required for evaluating children [2, 14]. In this study, we focused on upper extremity function and chose to translate the short form of the PROMIS Parent Proxy Upper Extremity Short Form 8a to make it easier and quicker for use and evaluation in outpatient clinics.

Despite the usefulness of the test, no Thai translation has been performed to help Thai patients understand the questions and complete the form correctly. Our study’s purpose was to develop a Thai translation of this form and test its reliability and validity in Thai children with congenital upper extremity anomalies.

## Methods

This cross-sectional study was approved by the Institutional Review Board. The agreement and permission to translate the PROMIS Pediatric and Parent Proxy Short Form v2.0 - Upper Extremity 8a items into Thai was requested from the PROMIS Health Organization (PHO), which is a copyright holder. After receiving approval from the PHO, the process of cross-cultural translation was performed.

### Phase 1: Questionnaire translation

All items, item contexts, and answer options in the PROMIS Pediatric Upper Extremity Short Form 8a version 2 (Ped-UE-SF) and Parent Proxy were translated using the Functional Assessment of Chronic Illness Therapy (FACIT) translation methodology. This methodology was employed in the translation of all PROMIS adult and paediatric items and is consistent with the guidelines recommended by the International Society for Pharmacoeconomic and Outcomes Research (ISPOR) for the translation of patient-report outcome instruments [15–17]. First, the Thai version of the PROMIS Ped-UE-SF 8a items was translated. The details of each translation step were as follows.

1) Two simultaneous forward translations by professional translators who are native Thai speakers were performed.

2) The reconciled single Thai translation was performed by a third translator who selected one of the forward translations (for Items 1–4 and Items 6–7) or created a hybrid version (for Items 5 and 8).

3) A native English-speaking translator who is fluent in Thai language conducted a back-translation of the reconciled version.

4) The back-translation was reviewed by the Translation Project Manager (TPM), who compared sources and back-translated English versions to identify discrepancies in the back translation and harmonization between the languages.

5) Three hand surgeons who are native speakers of Thai language conducted expert reviews and selected the most appropriate translation for each item or provided alternate translations if the previous translations were not acceptable. The reconciled versions from forward translations (Items 1–4 and Items 6–7) were selected. For items 5 and 8, the hybrid versions were created from forward and reconciled translations.

6) In a pre-finalization review, the TPM evaluated the reviewers’ comments, identified potential problems in their recommended translations, and formulated questions and comments to guide the language coordinator regarding the target language.

7) A Language Coordinator (LC), who is a native Thai speaker, determined the final translation by reviewing all of the information in the Item History and addressing the TPM’s comments.

8) Harmonization and quality was assured by the TPM who made a preliminary assessment of the accuracy and equivalence of the final translation. All documents and translation history were sent for quality review and approval by the PROMIS Statistical Center.

9) The formatting, typesetting and proofreading of the final questionnaire and items were conducted by two proofreaders who reconciled any comments.

10) Cognitive testing and linguistic validation of the Thai language version was pretested with children who were native Thai speakers. The goal was to verify that the meaning of each item was equivalent to the English source after translation.

11) An analysis of participants’ comments and finalization of the translation were conducted. The TPM compiled participants’ comments and summarized the issues. The LC reviewed the issues and proposed translation solutions. The TPM verified that solutions proposed by the LC harmonized with the source material and with other languages.

After the Thai version of the PROMIS Ped-UE-SF 8a items was translated by standard FACIT translation methodology and certified by the PHO, then, the parent proxy version was translated as follows:

- Replacing “I” with “My child” and making the appropriate modifications for the third person.
- Providing a cognitive debrief of the parent proxy version with parents of children aged 5 to 17 years.
- Analysing comments from parents for each item.
- Finalizing the translation while maintaining consistency between the paediatric self-report and parent proxy versions.

### Phase 2: Psychometric validation

After certification by the PHO, the Thai version of the PROMIS Parent Proxy UE-SF 8a items was used to evaluate Thai children with different types of congenital upper extremity anomalies, along with the Thai version of the MHQ [8]. All questionnaires were completed by the children’s parents. Informed consent was obtained from all participants. Demographic data, including age, sex, dominant hand, affected limbs, type of anomaly conditions, parent occupations and how bills are paid, were collected. At the first visit, parents were asked to complete the PROMIS questionnaire and the MHQ on the paper forms at the outpatient clinic. After they finished both questionnaires, the parents were given a copy of the PROMIS questionnaire to keep with them. After 1 week, the parents were asked to complete the same PROMIS questionnaire and return it to the clinic. The score of each item from the questionnaires was collected. The reliability and validity of the Thai-PROMIS parent Proxy UE-SF 8a items and the Thai-MHQ were analysed.

### Statistical analysis

The demographic data were reported in terms of means and standard deviations. To evaluate the reliability and validity of the Thai-PROMIS Parent Proxy UE-SF 8a questionnaire, the test and retest reliability were measured with the intraclass correlation coefficient (ICC), which can range from 0 to 1. If the ICC was > 0.7, good reliability was indicated [18]. The internal consistency reliability was evaluated by Cronbach’s alpha coefficient. Scores between 0.7 and 0.8 were considered acceptable, 0.8 and 0.9 represented good reliability and > 0.9 indicated excellent reliability [19]. Pearson’s correlation coefficients were used to evaluate the construct validity of the correlation between the Thai-PROMIS UE-SF 8a and Thai-MHQ. The level of correlation was rated as weak (r = 0.10–0.39), moderate (r = 0.40–0.69), or strong (r = 0.70–0.89) [20]. All statistics were calculated with IBM SPSS Statistics Version 14.0.

## Results

The PROMIS Pediatric and Parent Proxy Upper Extremity Short Form 8a items version 2 were translated by following all 11 steps of the FACIT translation methodology and certified by the PROMIS Health Organization. During the translation process, 3 expert reviewers who are hand surgeons and native Thai speakers provided comments as follows. For Item 3, “I can open the rings in school binders”, all expert reviewers recommended clarifying “the rings in school binders” in a clearer term for children to understand. Therefore, we added an explanation of the rings by adding the word “metal rings” and used the word “document binders” instead of “school binders” for easier understanding by Thai children. Therefore, the back translation of the precognitive debriefing test version was “I can open the metal rings in document binders”. For Item 4, “I can pour a drink from a full pitcher”, some expert reviewers commented on using the word “water” instead of “a drink” for easier understanding by young children. However, in the final version, we decided to use the word “a drink” because “a drink” can include all types of beverages, including water.

Cognitive debriefing of the Thai-PROMIS Ped-UE-SF 8a was performed for 5 children, and the parent proxy version was tested with 5 parents of children aged 5 to 17 years. The comments for 8 items during the process of cognitive debriefing are shown as follows (Table 1):

**Table 1:** Cognitive debriefing of the Thai PROMIS Paediatric and Parent Proxy Upper Extremity Short Form 8a

Item	Paediatric	Parent-proxy	Comment
1	I could button my shirt or pants	My child could button his/her shirt or pants	• No comment
2	I could open a jar by myself	My child could open a jar by himself/herself	• No comment
3	I could open the rings in school binders	My child could open the rings in school binders	• One child advised to emphasize the word “open the rings” to avoid misunderstanding between “open the rings” or “open only the front cover of the school binders”.
4	I could pour a drink from a full pitcher	My child could pour a drink from a full pitcher	• The size and weight of the pitcher might affect the ability of a child to perform this task.
5	I could pull a shirt on over my head by myself	My child could pull a shirt on over his/her head without help	• Different types and sizes of T-shirts might affect the difficulty of this activity.
6	I could pull open heavy doors	My child could pull open heavy doors	• All children and parents understood the meaning of the activity that was asked (to pull the door open). • However, the children and parents asked questions regarding how heavy the door was. • Three children were concerned that if the door was very big and heavy, they would not be able to pull it open. • They answered this question with “With a little trouble” (2 children) or “With some trouble” (1 child). • The parents were also concerned about how heavy the door was. If the door was very heavy, the children might not be able to pull it open. • After reviewing the original English item, it does not specify the weight of the door. • The English item leaves it up to interpretation what the respondent considers to be “heavy”. • We decided not to insert a weight or any example in the translation because it would not help children much and would deviate from the original source.
7	I could put on my shoes by myself	My child could put on his/her shoes without help	• All children understood that the word “shoes” meant the type of shoes that they need to put their feet inside. • Emphasized that the shoes mentioned in this item should not be the sandals which is much easy to wear.
8	I could use a key to unlock a door	My child could use a key to unlock a door	• No comment

This Thai version of the PROMIS Parent Proxy UE-SF 8a was used to evaluate upper extremity function in 30 Thai children with congenital upper extremity anomalies. The mean age of the children was 4.47 ± 2.08 years (range 1–9 years old). All questionnaires were completed by the children’s parents. There were 18 boys (60%) and 12 girls (40%) in this study. Most of the children’s problematic hands were their right hands (Right: Left: Both hands = 14: 7: 9), which was also the dominant hand in most children (Right: Left: Both hands = 18: 11: 1). Most children in this report were in elementary school grades 1–3. Most children had a family income of approximately 25,000 to 35,000 Thai baht (approximately $690–830 US Dollars) per month and had universal coverage for paying bills.

There were several types of upper extremity anomalies in this study. The main diagnosis was thumb duplication in 11 patients (36.7%), followed by syndactyly (4 children, 13.3%), congenital trigger thumb (3 children, 10%) and obstetric brachial plexus palsy (3 children, 10%). Due to the young age of the children in this study, all questionnaires were completed by the parent proxies for the children. The upper extremity function of these children was evaluated by the Thai-PROMIS Parent proxy UE-SF (tests and retests 1 week later) and the Thai-MHQ. The mean time to complete the PROMIS questionnaire was 164.23 ± 22.58 seconds (range 102–204), while for the MHE questionnaires, it was 337.8 ± 49.37 seconds (range 246–435). The score of each patient and each test are shown in Table 2.

**Table 2:** Total score and correlation between the Thai-PROMIS Parent Proxy UE-SF 8a and Thai-MHQ in children with congenital upper extremity anomalies (n = 30)

				Thai-PROMIS UE-SF 8a		Thai-MHQ	MHQ 6 Domains (Total 100 points per domain)					
ID.	Age	Type of anomalies	Affected hand	Test (40 points)	Retest (40 points)	Total MHQ (100 points)	1. Hand function	2. Activity	3. Work performance	4. Pain	5. Aesthetics	6. Satisfaction
1	4	Amniotic band	Both	23	25	62.52	67.50	57.62	0.00	0.00	62.50	87.50
2	8	Ulnar deficiency	Left	16	18	61.33	70.00	79.64	60.00	25.00	50.00	33.33
3	2	Obstetric brachial plexus palsy	Right	24	24	74.60	100.00	51.79	75.00	0.00	50.00	70.83
4	1	Thumb duplication	Right	22	22	64.26	65.00	39.29	0.00	0.00	93.75	87.50
5	5	Ulnar Polydactyly	Right	35	36	87.01	75.00	85.00	85.00	0.00	81.25	95.83
6	6	Syndactyly (complex)	Both	17	19	50.70	45.00	15.48	50.00	0.00	43.75	50.00
7	4	Thumb duplication	Left	15	16	62.08	90.00	92.50	15.00	25.00	50.00	50.00
8	3	Thumb duplication	Right	25	26	76.11	100.00	70.00	95.00	0.00	37.50	54.17
9	4	Amniotic band	Both	20	20	91.28	85.00	86.67	100.00	0.00	78.13	97.92
10	1	Obstetric brachial plexus palsy	Left	8	8	46.04	50.00	7.50	0.00	0.00	43.75	75.00
11	8	Thumb duplication	Right	40	40	60.28	80.00	100.00	35.00	20.00	0.00	66.67
12	5	Thumb duplication	Both	40	40	86.20	75.00	85.95	75.00	0.00	87.50	93.75
13	3	Obstetric brachial plexus palsy	Right	26	27	92.64	100.00	60.00	100.00	0.00	100.00	95.83
14	2	Congenital trigger thumb	Left	20	19	84.46	90.00	16.79	100.00	0.00	100.00	100.00
15	4	Radial deficiency	Right	23	26	55.67	90.00	43.21	55.00	0.00	0.00	45.83
16	3	Thumb duplication	Right	29	32	92.74	100.00	61.43	95.00	0.00	100.00	100.00
17	5	Congenital trigger thumb	Both	39	39	74.42	100.00	87.38	30.00	12.50	62.50	79.17
18	3	Thumb duplication	Left	28	29	84.03	75.00	37.50	100.00	0.00	100.00	91.67
19	6	Syndactyly (complex)	Both	26	26	69.96	57.50	56.43	100.00	15.00	50.00	70.83
20	5	Thumb duplication	Right	36	38	97.70	100.00	90.36	100.00	0.00	100.00	95.83
21	8	Ulnar Polydactyly	Right	39	40	83.87	100.00	98.21	5.00	0.00	100.00	100.00
22	5	Radial deficiency	Both	20	21	51.38	42.50	52.86	35.00	20.00	37.50	60.42
23	4	Thumb duplication	Left	33	33	91.24	90.00	72.86	95.00	0.00	93.75	95.83
24	2	Syndactyly (complex)	Both	8	8	28.32	10.00	7.86	0.00	0.00	12.50	39.58
25	3	Congenital trigger thumb	Both	25	26	88.95	90.00	57.86	90.00	0.00	100.00	95.83
26	5	Thumb duplication	Right	39	39	98.00	100.00	92.14	100.00	0.00	100.00	95.83
27	7	Clasped thumb	Right	38	38	93.00	100.00	89.64	85.00	0.00	87.50	95.83
28	5	Hypoplastic thumb	Right	28	30	84.34	100.00	61.43	80.00	0.00	81.25	83.33
29	9	Thumb duplication	Right	40	40	98.96	100.00	100.00	100.00	0.00	93.75	100.00
30	4	Syndactyly (simple)	Left	31	30	86.29	85.00	68.57	85.00	0.00	87.50	91.67
Correlation				ICC		Pearson’s Correlation						
				0.9909		0.6999	0.6195	0.7432	0.3841	-0.1473	0.4262	0.5905

To evaluate the reliability and validity of the Thai-PROMIS Parent Proxy UE-SF 8a, the test-retest reliability evaluated by ICCs of Thai-PROMIS Item 1–8 were 0.9906, 0.9650, 0.9425, 0.9890, 0.9636, 0.9538, 0.9488 and 0.9482, respectively, and 0.9909 for the total, which indicated good reliability. When comparing the internal consistency of this translated questionnaire, the Cronbach’s alpha of all items was 0.923, and after excluding each item, it was more than 0.9 (ranging from 0.9056–0.9253), representing excellent reliability. The Pearson’s correlation coefficient between the Thai-PROMIS Parent Proxy UE-SF 8a and Thai-MHQ is shown in Table 2. Regarding each domain, a strong correlation was found in Domain 2, which evaluated activities of daily living (r = 0.7432); a moderated correlation was found for Domain 1 (overall hand function, r = 0.6195), Domain 5 (aesthetics, r = 0.4264) and Domain 6 (satisfaction, r = 0.5905); and a weak correlation was found for Domain 3 (work performance, r = 0.3841) and Domain 4 (pain, r = -0.1473). Finally, the Thai version of the PROMIS Parent Proxy UE-SF-8a showed a moderate correlation with the overall Thai-MHQ score (r = 0.699) (Table 2).

## Discussion

The PROMIS Parent Proxy Upper Extremity Short Form 8a version 2 is one of the most commonly used items to help report the parental aspect of patients under 8 years old. It comprises an 8-item bank that parents can fill on their own and provides a physical function score ranging between 0 and 100. The paediatric PROMIS Physical Function assessment is the combination of individual mobility and upper extremity assessments that establish separate component scores. PROMIS scores are normalized to a mean score of 50 and a standard deviation of 10, with a theoretical range of 0 to 100. A higher score corresponds to better physical function. In this study, we developed the Thai version of the PROMIS Pediatric Upper Extremity Short Form 8a items and a parent proxy that was specifically designed to evaluate the upper extremity function of children with specific languages that match those of young children and parents with young children. The short-form questionnaire is advantageous because it requires less time to complete, has less complicated data collection and is easily understood by participants, especially in busy outpatient clinics.

To test the quality of this questionnaire, the Thai-PROMIS Parent Proxy UE-SF was used to evaluate children with various types of congenital upper extremity problems and different levels of hand function. In this study, all questionnaires were completed by the parents of these children. To test the reliability, we compared the Thai-PROMIS UE-SF with the Thai-MHQ. We chose the Thai-MHQ because this questionnaire has 6 domains for evaluation (overall hand function, activities of daily living, work performance, pain, aesthetics and satisfaction). The results showed that the Thai-PROMIS UE-SF had the best correlation with Domain 2, which determines the assessment of upper extremity function. Moreover, this newly translated questionnaire showed good internal consistency and excellent reliability (test-retest ICC = 0.9909 and Cronbach’s alpha = 0.923). After administering this questionnaire to 30 children, we found that the Thai-PROMIS Parent Proxy UE-SF was easy to use and less time-consuming than the Thai-MHQ (the mean questionnaire completion time was 164.23 ± 22.58 s vs. 337.8 ± 49.37 s, respectively). Our results align with those of a previous report on an English language questionnaire that showed high convergent validity between the upper extremity domains of the PROMIS and PODCI, with a lower time to completion for the PROMIS short-form [21, 22].

There were some limitations in this study. First, all children were young and needed a parent proxy to perform the evaluation. We did not have the information of testing the paediatric version that should be done by the children themselves. However, during the process of translation, we followed the standard protocol and performed cognitive debriefing with 5 children. All children responded that they clearly understood our Thai translation of the questionnaire. However, in the future, we will test the reliability of this questionnaire in older children. Second, the time between the test and retest may have been too short; hence, some participants may have remembered their previous answers and tended to answer the questions in the same way. Third, the sample size in this study was small due to restrictions during the COVID-19 pandemic. Last, the study was performed based on self-reported measures and questionnaires, which are inclined to have a response bias.

## Conclusions

The Thai version of the PROMIS Parent Proxy Upper Extremity Short Form 8a version 2 was translated with standard FACIT translation methodology and tested for reliability and validity in children with upper extremity anomalies. This translated questionnaire was a valid, reliable and easy-to-use patient-reported outcome measure that can help to assess function in children with congenital upper extremity anomalies.
